# New recommendation and coverage of low-dose computed tomography for lung cancer screening: uptake has increased but is still low

**DOI:** 10.1186/s12913-018-3338-9

**Published:** 2018-07-05

**Authors:** Jiang Li, Sukyung Chung, Esther K. Wei, Harold S. Luft

**Affiliations:** 1 Palo Alto Medical Foundation Research Institute, 795 El Camino Real, Ames Building, Palo Alto, CA 94301 USA; 2 California Pacific Medical Center Research Institute, Sutter Health Affiliate, 475 Brannan St #220, San Francisco, CA 94107 USA

**Keywords:** Implementation, Multilevel analysis, Cancer prevention and early detection, Preventive services, Health policy change

## Abstract

**Background:**

In 2013, the US Preventive Services Task Force (USPSTF) issued recommendations for low-dose computed tomography for lung cancer screening (LDCT-LCS), but there continues to be a dearth of information on the adoption of LDCT-LCS in healthcare systems. Using a multilevel perspective, our study aims to assess referrals for LDCT-LCS and identify facilitators and barriers to adoption following recent policy changes.

**Methods:**

A retrospective analysis of electronic medical record data from patients aged 55–80 years with no history of lung cancer who visited a primary care provider in a large healthcare system in California during 2010–2016 (1,572,538 patient years). Trends in documentation of smoking history, number of eligible patients, and lung cancer screening orders were assessed. Using Hierarchical Generalized Linear Models, we also evaluated provider-level and patient-level factors associated with lung cancer screening orders among 970 primary care providers and 12,801 eligible patients according to USPSTF guidelines between January 1st, 2014 and December 31st, 2016.

**Results:**

Documentation of smoking history to determine eligibility (59.2% in 2010 to 77.8% in 2016) and LDCT-LCS orders (0% in 2010 to 7.3% in 2016) have increased since USPSTF guidelines. Patient factors associated with increased likelihood of lung cancer screening orders include: younger patient age (78–80 vs. 55–64 years old: OR, 0.4; 95% CI, 0.3–0.7), Asian race (vs. Non-Hispanic White: OR, 1.6; 95% CI, 1.1–2.4), reported current smoking (vs. former smoker: OR, 1.7; 95% CI, 1.4–2.0), no severe comorbidity (severe vs. no major comorbidity: OR = 0.2, 95% CI = 0.1–0.3; moderate vs. no major comorbidity: OR = 0.5; 95% CI = 0.4–0.7), and making a visit to own primary care provider (vs. other primary care providers: OR, 2.4; 95% CI, 1.7–3.4). Appropriate referral for lung cancer screening varies considerably across primary care providers. Provider factors include being a female physician (vs. male: OR, 1.6; 95% CI, 1.1–2.3) and receiving medical training in the US (foreign vs. US medical school graduates: OR = 0.4, 95% CI = 0.3–0.7).

**Conclusions:**

Future interventions to improve lung cancer screening may be more effective if they focus on accurate documentation of smoking history and target former smokers who do not regularly see their own primary care providers.

## Background

Lung cancer is the leading cause of cancer-related death for Americans [1]. The National Lung Screening Trial (NLST) demonstrated that among individuals with a high risk of lung cancer, a 20% relative reduction in lung cancer mortality was observed with low-dose computed tomography for lung cancer screening (LDCT-LCS) compared to chest X-ray [2]. Those results formed the basis of the current screening recommendations adopted by almost all major organizations [3–7]. In December 2013, the US Preventive Services Task Force (USPSTF) released a recommendation for LDCT-LCS for adults 55–80 years of age who have a 30 pack-year smoking history and currently smoke, or those who have quit within the past 15 years [3]. Beginning February 2015, LDCT-LCS became a Medicare-covered preventive service for eligible beneficiaries [8]. Under Section 2713 of the Affordable Care Act (ACA) [9], private health plans must provide coverage for annual lung cancer screening for eligible adults aged 55–80 years and may not impose cost-sharing (such as copayments, deductibles, or co-insurance) on patients. New recommendations issued by the USPSTF are required to be covered without cost-sharing beginning in the plan year that begins on or after exactly 1 year from the latest issue date. As the only procedure proven to reduce lung cancer mortality in this high-risk population, implementation of LDCT-LCS could reduce mortality.

While the recent policy changes provide guidelines for screening that should lead to identifying early-stage lung cancer, it is unknown how screening is implemented in real-world clinical care. Data are being collected via the national Lung Cancer Screening Registry, a CMS-approved clinical practice registry gathering information from all patients who undergo LDCT-LCS, and these data will ultimately provide the opportunity to learn about lung cancer screening. Development of the registry, however, is still underway and may take several years to become publicly available [10]. Importantly, the registry tracks patients who are screened only, not those who might be eligible and are not screened. One recent Veterans Health Administration (VHA) study reported low screening rates overall, and several challenges in implementing LDCT-LCS including difficulties identifying patients who were eligible for screening and coordinating the screening and post-screening follow-up processes [11].

Research exploring factors associated with lung cancer screening is still nascent, with early qualitative work exploring perceptions of lung cancer screening among smokers [12–15] and health care providers [16, 17] and focusing on intention to screen rather than actual screening behavior. The clinical trials such as the NLST enrolled a relatively healthy population (e.g., younger, more likely to be former smokers) whose adherence was high [18], and with those declining trial participation described being too old to benefit [15]. Studies examining relationship between smoking status and participation in lung cancer screening showed conflicting results. One large study of nonrandomized lung cancer screening of smokers [19] reported, the self-selected screening participants tended to be current smokers, have smoked a greater number of pack-years, and more likely to have a family history of lung cancer than nonparticipants, whereas another clinical trial identified current smoking as a barrier to trial uptake among high-risk individuals declining participation [20]. Furthermore, the severity of comorbidities among elderly patients may influence LDCT-LCS referrals as potential benefit of screening is minimal for people with major health problem that substantially limits life expectancy or the ability to have curative lung surgery [2]. A positive patient-provider relationship also has been identified as potentially influential in the shared decision making process in cancer screening [21, 22]. Our aim is to characterize and understand the current uptake of lung cancer screening after the policy changes, and describe existing factors associated with LDCT-LCS referrals.

Within a large community healthcare setting in northern California serving a diverse patient population with varying insurance types, we examined the implementation of the USPSTF recommendations for LDCT-LCS following a series of guideline and reimbursement policy changes. The purpose of the study is twofold: a) to assess the changes in documentation of smoking history, number of eligible patients, and medical orders for LDCT-LCS between 2010 and 2016, and (b) to explore patient- and provider-level factors associated with medical orders for LDCT-LCS between 2014 and 2016 (up to 3 years after the 2013 USPSTF recommendation). To our knowledge, this is the first study of these factors in this type of setting.

## Methods

### Study population

A retrospective analysis was performed using electronic health records (EHR) data from patients in a large community healthcare system located in northern California. Unlike most prepaid group practices or HMOs, patients in this healthcare system are covered by a multitude of payers. For example, among patients who were aged between 55 and 80 and who used primary care services at the largest medical foundation in this healthcare system in 2014, primary insurances were: Commercial FFS/PPO 45%, Medicare FFS 35%, Commercial HMO 12%, Medicare HMO 5%, and other insurances or self-pay (uninsured) 3%. Patients had to be between 55 and 80 years old and have had at least one office visit to a Family Medicine or Internal Medicine provider between January 1st, 2010 and December 31st, 2016, and no evidence of lung cancer. This study was approved by the organization’s Institutional Review Board.

**Fig. 1:**
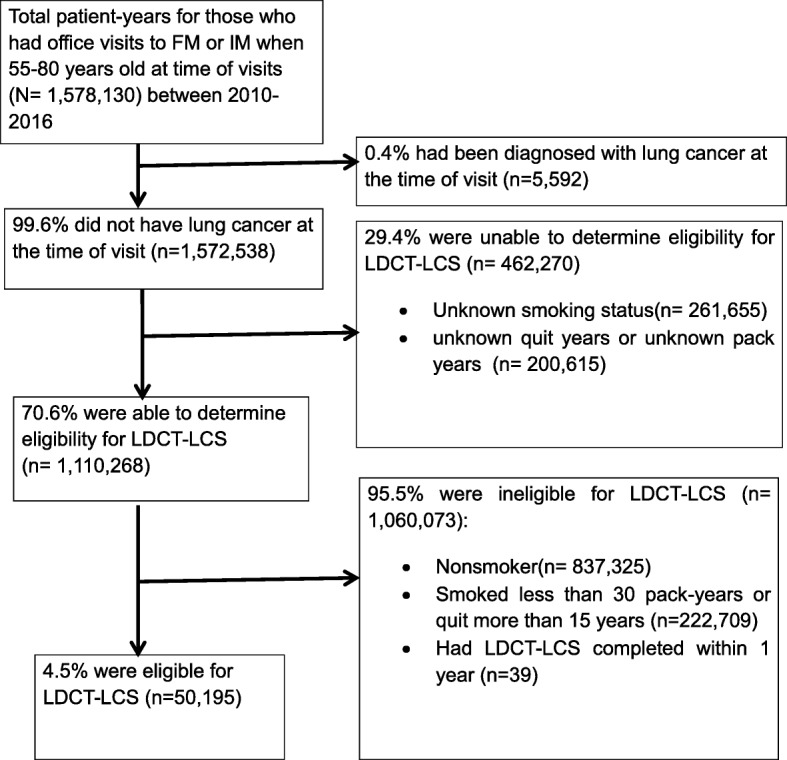
Consort diagram for eligibility determination for low-dose computed tomography for lung cancer screening (LDCT-LCS)

In the first part of the study, a patient-year was the unit of analysis. Because the guidelines recommend annual screening, we examine each year separately, so a patient could appear in the denominator (and numerator) in multiple years. In each year, we examine a patient’s smoking history information as represented in his or her EHR that year, without imputation of missing values using documented smoking history in the earlier or later years. Figure 1 shows the categorization of patient years during 2010–2016. Of those with no evidence of lung cancer (*n* = 1,572,538 patient years over 7 years), 261,655 patient-years had an unknown smoking status (i.e., no indication of current/former/non-smoker). For many visits with a known smoking status, the medical record did not contain enough information to determine patient eligibility by calculating the number of “pack years” they had smoked or the quit year if they had quit smoking (*n* = 200,615 patient-years). Excluding those cases in which we were unable to determine eligibility for LDCT-LCS, 70.6% (*n* = 1,110,268 patient-years) had at least one visit with documented smoking history sufficient to determine eligibility for LDCT-LCS in that year. Of those with sufficient information, 4.5% (*N* = 50,195 patient-years) represented patients eligible for annual LDCT-LCS based on the guidelines. The reasons for not being eligible for LDCT-LCS include: being a nonsmoker (*n* = 837,325 patient-years); smoking less than 30 pack-years or quit more than 15 years (*n* = 222,709 patient-years); and having had LDCT-LCS completed within 1 year (*n* = 39 patient-years).

In the second part of the study, the patient was the unit of analysis. Patients meeting all of the USPSTF LDCT-LCS guideline criteria (no signs or symptoms of lung cancer; history of cigarette smoking of at least 30 pack-years; and currently smoking or quit within the previous 15 years) at any office visit to a Family Medicine or Internal Medicine provider after the 2013 USPSTF recommendation were considered eligible for LDCT screening and included in the multilevel analysis.

The final sample for multilevel analysis included 12,801 patients who were eligible for LDCT-LCS and 970 primary care providers (PCP) between January 1st, 2014 and December 31st, 2016. Patients were excluded if their medical records did not include sufficient information to calculate the number of “pack years” or the year they had quit smoking. Information on smoking history, procedure orders, and physician billings were extracted for individuals included in the sample.

### Measurement

In the overall study sample (1,572,538 patient years over 2010–2016), we used several measures to describe the implementation of guideline-based LDCT-LCS each year: (1) Annual rate of documentation of smoking history is the proportion of patients who had documented smoking history among those with at least one visit in the year. (2) Annual rate of eligibility of patients for LDCT-LCS is the proportion of patients in a given year whose smoking history and other criteria met those of the guidelines for LDCT-LCS among those *with documentation of smoking history and at least one visit*. (3) Annual rate of receiving LDCT-LCS orders is the proportion of patients who received LDCT-LCS orders among those eligible for LCS.

In the multilevel analysis using data from 970 PCPs and 12,801 unique patients who were eligible for LDCT-LCS *at some point* during 2014–2016, the dependent variable was having received one or more LDCT-LCS orders (Yes/No) during 2014–2016. For each patient, patient demographics (age, sex, race/ethnicity, language preference), clinical characteristics (smoking status, smoking history, severity of major comorbidities, and visiting his/her own PCP) at the visit in which the first LDCT-LCS order was made (or last visit in the data if no LDCT-LCS order), the frequency of office visits during the calendar year, and calendar year were controlled for in the models. For the severity of major comorbidities, Charlson Comorbidity Index (CCI) [23] was used and patients were divided into four groups: no major comorbidity, with CCI scores of 0; mild, with CCI scores of 1–2; moderate, with CCI scores of 3–4; and severe, with CCI scores≥5. A patient’s own PCP refers to the PCP whom the patient actively chose when first visiting a PCP and usually goes to when he or she needs preventive services. Provider-level factors include sex, physician (vs. non-physician clinician), and where the PCP received their medical degree (in the US or outside of US).

### Analysis

First, we assessed annual trends in factors that are precursors to LDCT-LCS. For patients in the appropriate age range, we examined the rates at which smoking history was documented in the medical record; then among those patients, LDCT eligibility; and then the annual rate of medical orders for LDCT-LCS. We then assessed, among those for whom LDCT-LCS was recommended by the guideline, the bivariate relationship between having a medical order for LDCT-LCS (Yes/No) and patient demographic and clinical characteristics and characteristics of the patient’s provider. Provider-level variation was also assessed in terms of the percent of the PCP’s eligible patients who received an order for LDCT-LCS. For this last analysis, we limited the sample to providers who had at least five different eligible patients during 2014–2016.

To estimate multilevel factors associated with a LDCT-LCS order, data from 2014 to 2016 were analyzed using a multilevel structure with patients (level 1) nested within providers (level 2). A series of Hierarchical Generalized Linear Models (HGLM) using SAS PROC GLIMMIX were built to estimate odds ratios for the predictors and the random effect variance. The following three random intercepts models were estimated in a stepwise fashion. Model 1 is a null model including no fixed effects to examine intra-class correlations (ICC) apportioning the variance in the outcome across different levels. Model 2 includes only patient-level variables. Model 3 includes both patient and provider characteristics. Hypothesis tests for the fixed effects are based on Wald-type tests and the estimated variance-covariance matrix. The nested models were compared using a Likelihood Ratio Test. Statistical analyses were performed using SAS Version 9.3.

## Results

**Fig. 2:**
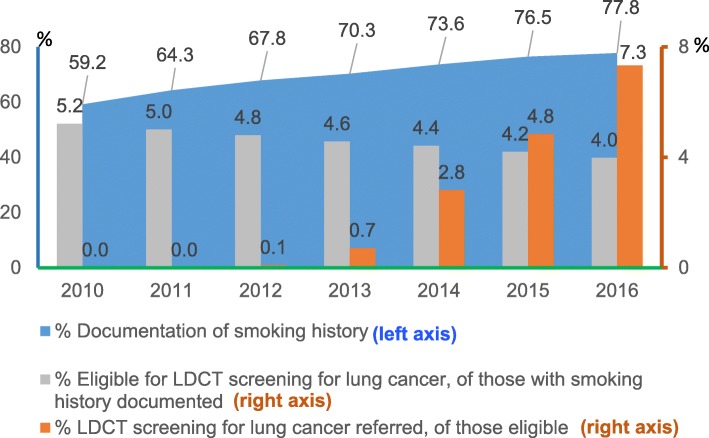
Trends in documentation of smoking history and referrals of lung cancer screening, 2010–2016

Among patients aged 55 and 80 years with no evidence of lung cancer and who had at least one office visit during the year to a Family Medicine or Internal Medicine provider, documentation of smoking history increased from 59.2% in 2010 to 77.8% in 2016 (Fig. 2). Of those with documentation of smoking history, the proportion of patients who were determined eligible for LDCT-LCS fell from 5.2% in 2010 to 4.0% in 2016. Of the eligible patients, the rate of receiving LDCT-LCS orders increased dramatically from 0% in 2010–2011 to 7.3% in 2016.

Of the 12,801 patients who were identified as eligible for LDCT-LCS, a total of 999 (7.8%) received one or more LDCT orders during 3 years (2014–2016) after the USPSTF recommendation was released (Table 1). Bivariate results show that the percent receiving LDCT-LCS orders varied significantly across Asian (11.1%), Hispanic (7.4%), Non-Hispanic White (7.4%), Black (7.0%), and other race/ethnicity groups (6.4%) (*p* = 0.04). Patients who were younger (i.e., 55–64 years old and 65–77 years old, compared to 78–80 years old), had less severe major comorbidities, made a visit to their own PCP, or were a current smoker were significantly more likely to receive an order (all *p* < 0.001). Patients with ≥60 pack year history of smoking were less likely to receive an order (*p* = 0.0004). Of the 970 PCPs, 63.3% were female, 79.4% was physician, and 17.6% graduated from medical schools outside of US.

**Table 1:** Characteristics of eligible patients for LDCT-LCS, 2014–2016 (*N* = 12,801)

	Having ever received LDCT-LCS order				*P* value
	Yes	%	No	%	
Age					<.0001
55–64	463	8.4	5027	91.6	
65–77	509	8.0	5859	92.0	
78–80	27	2.9	916	97.1	
Sex					0.71
Female	455	7.9	5303	92.1	
Male	544	7.7	6499	92.3	
Race					0.04
Hispanic	48	7.4	598	92.6	
Non-Hispanic White	748	7.4	9346	92.6	
Black	29	7.0	384	93.0	
Asian	52	11.1	415	88.9	
Other	23	6.4	339	93.6	
Language use					0.19
English	951	7.7	11364	92.3	
Non-English speaker	33	9.6	309	90.4	
Visit to own primary care provider					<.0001
Yes	887	8.5	9564	91.5	
No	109	4.7	2203	95.3	
Smoking status					<.0001
Current smoker	624	9.6	5846	90.4	
Former smoker	375	5.9	5956	94.1	
Pack-year (packs per day × years of smoking)					0.0004
30–39	399	8.6	4224	91.4	
40–49	307	7.8	3606	92.2	
50–59	149	8.3	1645	91.7	
60+	144	5.8	2327	94.2	
Severity of major comorbidities					<.0001
Severe (CCI ≥5)	36	2.2	1599	97.8	
Moderate (CCI =3–4)	112	5.1	2067	94.9	
Mild (CCI =1–2)	432	8.5	4644	91.5	
No major comorbidity (CCI =0)	419	10.7	3492	89.3	

**Fig. 3:**
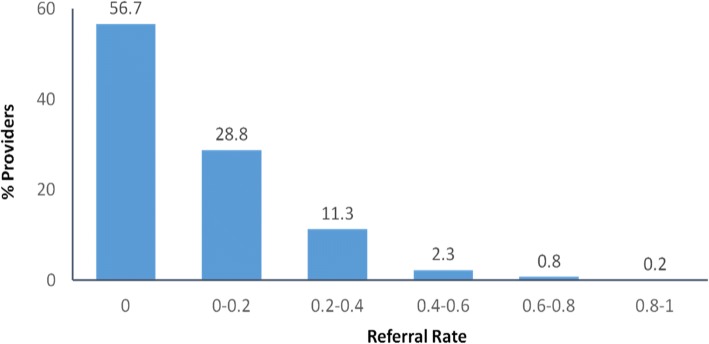
Average referral rates of lung cancer screening among 663 primary care providers, 2014–2016. Note: Providers (*n* = 307) with less than 5 eligible patients during 2014–2016 were excluded

There was substantial variation in referral rates across providers in this healthcare system (Fig. 3). A majority (56.7%) of providers made no referrals for eligible patients during the 3 years after the new USPSTF guideline. While under a third (28.8%) made some referrals, they did so for fewer than 20% of their eligible patients. Only 1% (*n* = 6) of providers referred more than 60% of their eligible patients.

Results from the HGLM (Table 2) also demonstrate substantial provider variation for LCS referral, as indicated by statistically significant Level-2 intercept (τ_00_ = 3.17; *p* < .0001) of the unconditional, null model (Model 1). Approximately 49% (ICC = .49) of the variability in the LDCT referral rate was accounted for by provider-level factors including but not limited to the provider factors observed in this study. Based on the likelihood ratio test, Model 2 with the patient-level independent variables being entered was a better fitting model than the unconditional, null model (Model 1). The addition of specific provider-level variables (Model 3) further improved model fit. In this final model, patients aged 78–80 years old were significantly less likely to receive lung cancer screening orders compared to those aged 55–64 years old (OR, 0.4; 95% CI, 0.3–0.7). Patients with severe (OR, 0.2; 95% CI, 0.1–0.3) or moderate (OR, 0.5; 95% CI, 0.4–0.7) major comorbidities were significantly less likely to receive an order than those without any major comorbidity. Asian patients were more likely to receive LDCT-LCS orders than non-Hispanic White patients (OR, 1.6; 95% CI, 1.1–2.4), current smokers were more likely to receive an order than former smokers (OR, 1.7; 95% CI, 1.4–2.0), and patients seeing their own PCP were also significantly more likely to receive an order (OR, 2.4; 95% CI, 1.7–3.4). Female providers were more likely to give an order for eligible patients (OR, 1.6; 95% CI, 1.1–2.3) while foreign medical school graduates were less likely (OR, 0.4; 95% CI, 0.3–0.7) to provide an order.

**Table 2:** Multilevel models for receiving medical order for lung cancer screening among eligible patients, 2014–2016 (*N* = 12,801)

Fixed Effects	Model 1	Model 2	Model 3^a^
	Estimate (SE)	Estimate (SE)	Estimate (SE)
Intercept	−3.56** (0.11)	−4.41*** (0.22)	−5.44*** (0.88)
Patient-level Factors		OR (95% CI)	OR (95% CI)
Age			
78–80		0.4**(0.3–0.7)	0.4**(0.3–0.7)
65–77		1.2 (1.0–1.4)	1.2 (0.9–1.4)
55–64		1.0	1.0
Sex			
Female		1.1 (0.9–1.3)	1.0 (0.8–1.2)
Male		1.0	1.0
Race			
Hispanic		1.2 (0.8–1.8)	1.1 (0.8–1.7)
Black		1.2 (0.8–2.0)	1.1 (0.6–1.9)
Asian		1.6* (1.1–2.4)	1.6* (1.1–2.4)
Other		1.0 (0.6–1.6)	0.9 (0.5–1.6)
Non-Hispanic White		1.0	1.0
Smoking status			
Current Smoker		1.6***(1.4–2.0)	1.7***(1.4–2.0)
Former Smoker		1.0	1.0
Pack-year (packs per day*years of smoking)			
60+		1.0 (0.8–1.3)	1.0 (0.8–1.4)
50–59		1.2 (0.9–1.6)	1.2 (0.9–1.6)
40–49		1.0 (0.9–1.3)	1.1 (0.9–1.3)
30–39		1.0	1.0
Visiting one’s own primary care provider			
Yes		2.4***(1.8–3.2)	2.4***(1.7–3.4)
No		1.0	1.0
Severity of major comorbidities			
Severe (CCI ≥5)		0.2***(0.1–0.3)	0.2***(0.1–0.3)
Moderate (CCI =3–4)		0.6***(0.4–0.8)	0.5***(0.4–0.7)
Mild (CCI =1–2)		1.0 (0.8–1.2)	1.0 (0.8–1.3)
No major comorbidity (CCI =0)		1.0	1.0
Provider-level Factors			OR (95% CI)
Gender			
Female			1.6* (1.1–2.3)
Male			1.0
Professional			
Physician			3.1 (0.6–16.1)
Other			1.0
Graduated from medical universities outside of US.			
Yes			0.4** (0.3–0.7)
No			1.0
Error Variance	Estimate (SE)	Estimate (SE)	Estimate (SE)
Level-2 Intercept	3.17 (0.37)***	3.28 (0.41)***	2.63 (0.36)***
Model Fit			
-2 Log Likelihood	5873.46	4934.42***	3882.77***

ICC = .49; Control variables of Model 2 and 3 not presented in the table include frequency of office visits, and calendar year

*OR* Odds Ratio, *SE* Standard Error, *95% CI* 95% Confidence interval

*p < .05; ** *p* < .01; ***p < .0001

^a^Best fitting model

## Discussion

Using EHR data from a large community healthcare system, we assessed changes in documentation of smoking history and orders for LDCT for lung cancer after the USPSTF released its recommendations. We found substantial variation across providers in referral for LDCT-LCS. Lower medical order rates were found for male providers and foreign medical school graduates, but even after taking into account these factors, we observed large, unexplained variation across providers. Our findings are consistent with a previous population-based study looking at PCP barriers to LDCT-LCS which found that only about half indicated that they knew LCS was recommended by the USPSTF [24]. Reported barriers to LCS among providers included the potential harms associated with LDCT-LCS, uncertainty about the benefit of LDCT-LCS, and questions about insurance coverage for LCS.

At the patient level, we found that younger age, Asian race, being a current smoker, having minimal severe comorbidities, and seeing one’s own PCP were positive predictors of getting LDCT-LCS orders. Previous studies exploring smokers’ attitudes and beliefs about LDCT-LCS in both US and European suggest that being too old to benefit, concerns about cost, inconvenience, perceived smoking-related stigma, fatalism, radiation exposure fears, and distrust of the healthcare system may constitute barriers to LDCT-LCS [12, 14, 15]. The maximum age limit of 77 set by CMS for reimbursement of annual LDCT for lung cancer screening [8] may in part explain the lower referral rates among the oldest age group (78–80 years old), as well as the decreasing relative benefit of screening and early detection with increasing patient age. We found a lower rate (5.8%) of referrals for patients with the most pack-years (≥60 pack-years) but smoking history did not retain a significant relationship with getting LDCT-LCS orders after controlling for all other independent variables.

We found that LDCT-LCS orders are much more likely to occur if the patient has seen his or her own PCP. This makes sense given the counseling necessary before LDCT-LCS. On an interpersonal level, trusting relationships shape whether physicians and patients are able to engage in good discussions on cancer screening [25]. Relationships between patients and their own PCP will increase the likelihood that LDCT-LCS counseling will take place.

In our data, orders for LDCT-LCS have increased since the USPSTF recommendations, but overall remain very low. This is consistent with findings from the National Health Interview Surveys where a significant but still low uptake of CT scans for lung cancer screening was observed in high-risk smokers (5.8% in 2015 vs. 2.9% in 2010, *P* < .001) [26]. A potential explanation for the low medical order rate in the NHIS data is the lack of documentation of smoking history enabling appropriate selection of high-risk individuals for screening [11]. Recommending and covering LDCT in a targeted, high-risk population is designed to minimize the burden of screening, including the need for confirmatory tests in those with false-positive results and the anxiety related to testing [27]. In our study, although documentation of smoking history improved substantially from 2010 to 2016 (from 59.2 to 77.8%), incomplete documentation of smoking history remains an area for improvement.

Even when smoking status is known, determining screening eligibility for those who are at a high risk of lung cancer may not be straightforward. Many PCPs may not be fully aware of the selection criteria for lung cancer screening and may not be prepared to refer patients for appropriate screening [28, 29]. Further, eligibility based on USPSTF guideline may be debatable. Recent studies have shown that selection for screening using an individualized risk assessment tool is superior (in terms of higher sensitivity and specificity) to the current eligibility criteria based on age and cumulative smoking exposure alone [30]. The feasibility of assessing individual risk during routine primary care visits is challenging but an important area to build additional knowledge.

Studies have shown that, from a physician’s perspective, implementation of the new guidelines is hindered by the lack of available staff time, financial factors and the complexity of the information presented [31]. PCPs practicing in New Mexico clinics reported concerns about the feasibility and appropriateness of implementing LDCT screening, including insufficient infrastructure, access barriers, and financial burdens for patients [16]. A 3-year demonstration project at the VHA contacted all patients deemed eligible for LCS to discuss screening and set up an appointment, but this required devoted staff members including a coordinator, significant time resources, a rigorous and clear implementation guide for healthcare providers, and electronic tools and database [11]. This resource-intensive approach may not reflect what happens when implementing LDCT-LCS in health care systems other than VHA. In the community healthcare system of this study, there had been minimal effort promoting or advertising lung cancer screening at the system level between 2014 and 2016. For example, there was an introduction of the new recommendations for lung cancer screening by pulmonologists at one primary care provider meeting at the largest medical foundation in this healthcare system.

Several limitations of this analysis must be noted. We relied on structured data from billing, procedure, ordering, and administrative records, but many important constructs remain unexamined. For example, we did not examine guideline non-adherence (i.e., referrals for LDCT screening in persons not meeting the USPSTF criteria). We lack information on specific factors influencing patient and provider decision-making including physicians’ verbal recommendations and patients’ preferences, which may be in part available in unstructured, free-text EHR notes. Similarly, the completion of LDCT-LCS warrants further investigation but the organization is not an HMO, it is difficult to know whether a patient receiving a referral obtained a LDCT-LCS from a provider outside the system. Furthermore, using data from a single healthcare organization with a generally highly-insured patient population may limit the generalizability of our findings. By focusing on the adoption and implementation of the guidelines among those who have access to a healthcare system, we reduce the influence of disparities in access in the patterns we observed. In addition, smoking rates in California are well below the national average [32].

## Conclusions

Three years after the inception of USPSTF recommendations and Medicare coverage, uptake of LDCT-LCS remains very low. Wider adherence to the guidelines will first require better documentation of smoking history to enable providers to determine patients’ eligibility for lung cancer screening. There is a wide variation across PCPs in a healthcare organization in the uptake of guideline-based practice, suggesting needs for system-wide efforts to facilitate appropriate LCS. Barriers and facilitators specific to lung cancer screening at the patient, provider, and system level need to be better understood to inform targeted interventions to improve the entire lung cancer screening process. The higher rates of orders for current smokers suggests that either providers or patients may see less need for LDCT-LCS if the patient had quit smoking many years before. This is an area worthy of further exploration. Organizational factors should also be explored including the support available to PCPs, workflows, and other “standard practices” that can facilitate appropriate use of LDCT-LCS. Future interventions to improve lung cancer screening should pay particular attention to reaching former smokers, as well as those who are not visiting his/her own PCP.

## Abbreviations

95% CI: 95% Confidence interval
ACA: Affordable Care Act
CCI: Charlson Comorbidity Index
CMS: Centers for Medicare & Medicaid Services
EHR: Electronic health records
HGLM: Hierarchical Generalized Linear Models
ICC: Intra-class correlations
LDCT-LCS: Low-dose computed tomography for lung cancer screening
NLST: National Lung Screening Trial
OR: Odds ratio
PCP: Primary care providers
USPSTF: US Preventive Services Task Force
VHA: Veterans Health Administration
